# Induced defense strategies of plants against *Ralstonia solanacearum*

**DOI:** 10.3389/fmicb.2023.1059799

**Published:** 2023-01-26

**Authors:** Haoqi Shi, Yong Liu, Anming Ding, Weifeng Wang, Yuhe Sun

**Affiliations:** ^1^Tobacco Research Institute, Chinese Academy of Agricultural Sciences, Qingdao, Shandong, China; ^2^The Graduate School, Chinese Academy of Agricultural Sciences, Beijing, China; ^3^Tobacco Breeding and Biotechnology Research Center, Yunnan Academy of Tobacco Agricultural Sciences, Kunming, China

**Keywords:** *Ralstonia* *solanacearum*, cell wall, PTI, ETI, cell wall integrity, structural defense

## Abstract

Plants respond to *Ralstonia solanacearum* infestation through two layers of immune system (PTI and ETI). This process involves the production of plant-induced resistance. Strategies for inducing resistance in plants include the formation of tyloses, gels, and callose and changes in the content of cell wall components such as cellulose, hemicellulose, pectin, lignin, and suberin in response to pathogen infestation. When *R*. *solanacearum* secrete cell wall degrading enzymes, plants also sense the status of cell wall fragments through the cell wall integrity (CWI) system, which activates deep-seated defense responses. In addition, plants also fight against *R*. *solanacearum* infestation by regulating the distribution of metabolic networks to increase the production of resistant metabolites and reduce the production of metabolites that are easily exploited by *R*. *solanacearum*. We review the strategies used by plants to induce resistance in response to *R*. *solanacearum* infestation. In particular, we highlight the importance of plant-induced physical and chemical defenses as well as cell wall defenses in the fight against *R*. *solanacearum*.

## Introduction

Plants are exposed to a variety of biotic and abiotic stresses during growth (Panstruga et al., 2009). These stresses affect plant growth and lead to severe reductions in the yield of cash crops. Without discussing the interactions between abiotic stresses and plants, here, we focus on the induction of resistance in plants to *R*. *solanacearum*. *R*. *solanacearum* are highly damaging soil-borne pathogens that can infect more than 250 species of plants, including Solanaceae (Peeters et al., 2013). In order to invade, *R*. *solanacearum* first secretes cell wall degrading enzymes to destroy the cell wall of the host cell, and then relies on the type III secretion system (T3SS) to transfer a variety of type III effector proteins (T3Es) to the host cell to make it susceptible to disease (Coll and Valls, 2013). These T3Es suppress the immune response of plants through a variety of molecular mechanisms. Landry et al. (2020) provide a good summary of the various types of *R*. *solanacearum* T3Es that have been reported and the immune responses they induce. Accordingly, plants have evolved two layers of immune systems to defend themselves against attacks from pathogenic bacteria (Jones and Dangl, 2006; Wan et al., 2021). They are pathogen-associated molecular patterns (PAMPs)-triggered immunity (PTI) and effector-triggered immunity (ETI). Plant cells first recognize PAMPs through pattern recognition receptors (PRRs), which activate PTI. To counteract PTI, the pathogen secretes numerous T3Es into the plant cell to inhibit the PTI response. However, intracellular receptors that have evolved in plants, NLRs, can detect the activity of intracellular T3Es and thus activate the ETI immune response, inactivating the T3Es (Chiang and Coaker, 2015; Cui et al., 2015; Wu et al., 2021). However, some effector proteins can also successfully inhibit ETI, rendering immunity ineffective (Rufián et al., 2018; Nakano et al., 2020). Recent findings refute previous conclusions that PTI and ETI act separately and demonstrate a complex interaction between PTI and ETI (Ngou et al., 2021; Yuan et al., 2021).

The activation of the two-layer immune system in plants initiates a series of molecular regulatory mechanisms at the cellular level. These regulatory mechanisms involve the deposition of plant callose, changes in cell wall composition, and the production of resistant metabolites to defend against the infestation and spread of pathogenic bacteria (Shaban et al., 2018). This review focuses on a summary of the strategies involved in inducing resistance in plants against *R*. *solanacearum*, with a view to providing a reference for *R*. *solanacearum* control.

## Differences in the colonization pathways of *Ralstonia solanacearum*

**Figure 1:**
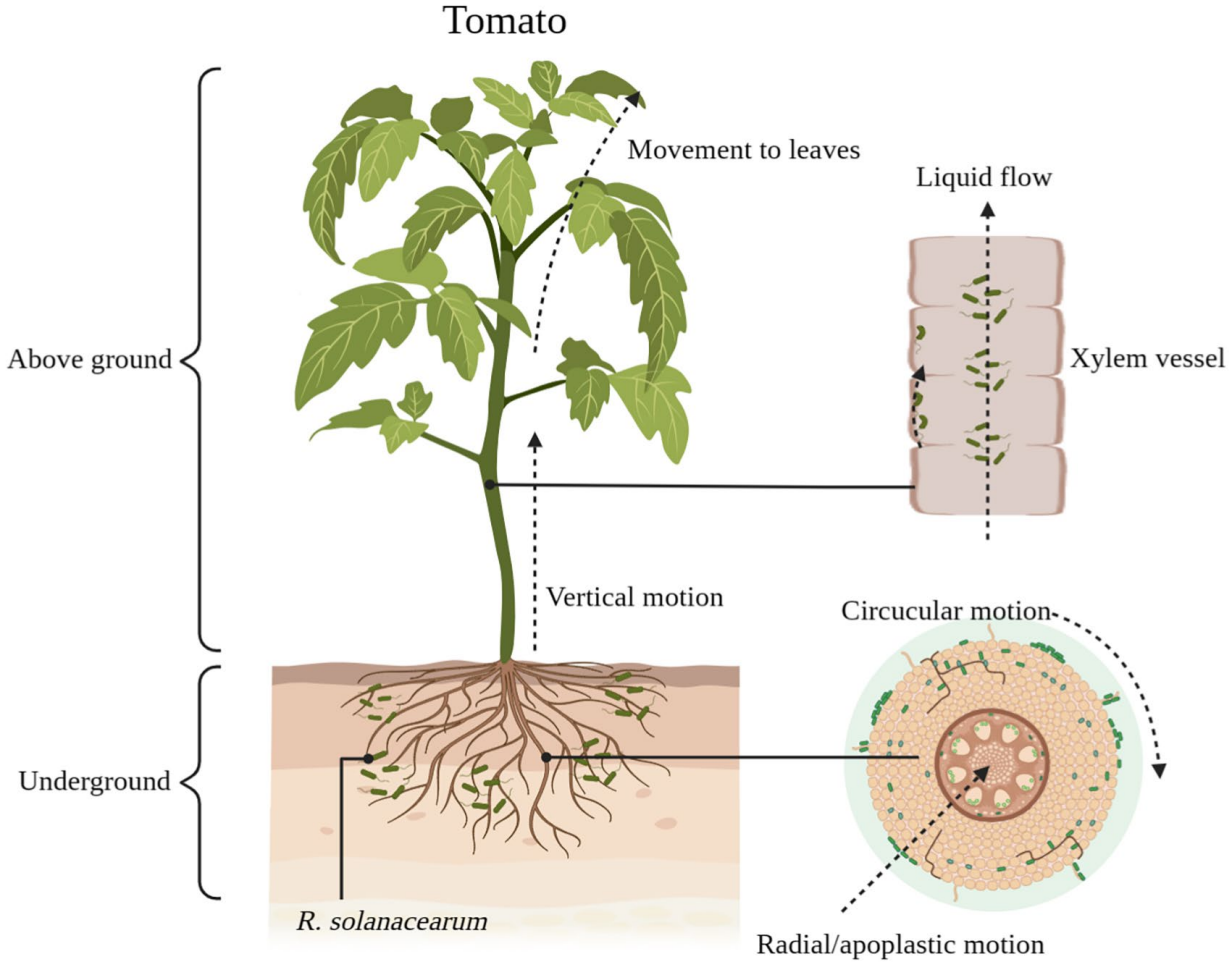
The process of *Ralstonia* *solanacearum* infesting tomatoes. *R*. *solanacearum* enter the xylem of tomatoes through the cell spaces of the roots. It gradually reaches the xylem through radial and circumferential movements before entering the xylem (lateral movement) and then moves vertically upward through the xylem vessel to the above-ground part of the tomato (longitudinal movement). Some of the *R*. *solanacearum* attach themselves to the xylem duct walls and use this area as an ecological niche for colonization, while others are suspended in the duct’s fluid stream up to the leaf area. Schematic diagram is drawn through BioRender (https://biorender.com/).

Green fluorescent protein markers make it easy to understand the colonization pathways of *R*. *solanacearum* in their hosts (Lowe-Power et al., 2018). Currently, the generally accepted pathway for *R*. *solanacearum* colonization is for *R*. *solanacearum* to enter the root cortex of the host and then reach the xylem through the intercellular space, where they proliferate and spread to the above-ground parts of the host (Figure 1; Bae et al., 2015). A portion of the *R*. *solanacearum* are planktonic in the sap flow of the host xylem, while another portion of the *R*. *solanacearum* use jerky movements to move along the walls of the vessel (Figure 1). These *R*. *solanacearum* eventually accumulate in the biofilm matrix, filling the entire duct and potentially impeding water flow, eventually causing the plant to wilt and die (Caldwell et al., 2017).

Although *R*. *solanacearum* successfully colonized different resistant plants, the time taken for colonization to reach the xylem from outside the roots varied. The time taken for *R*. *solanacearum* to reach the xylem from the root cortex was longer in resistant tomatoes than in susceptible tomatoes (Planas-Marquès et al., 2019). This difference was clearly observed by Caldwell et al. (2017) with the aid of scanning electron microscopy. In order to reveal this phenomenon of differential colonization, the researchers investigated the colonization of *R*. *solanacearum* at the tissue level in different resistant plants. The results showed that plant resistance to *R*. *solanacearum* acts in both roots and stems. In tomato-*R*. *solanacearum*; for example, resistance in tomato was associated with the ability to limit the spread of *R*. *solanacearum* from the root neck to the middle of the stem (Grimault and Prior, 1993; Nakaho et al., 2004).

In addition, grafting tests with resistant and susceptible roots/stems of tomato confirmed the role of both roots and stems in plant resistance (Planas-Marquès et al., 2019). A study by Planas-Marquès et al. (2019) further summarized that *R*. *solanacearum* movement and colonization were restricted by *R*. *solanacearum* at four tissue levels (root invasion, vertical upward movement to the stem, annular channels between vessel and radial diffusion of xylem to the pith/cortex) in *R*. *solanacearum*-resistant tomato. It has also been shown in tobacco that the mechanism of resistance to *R*. *solanacearum* in resistant tobacco is related to the ability to restrict *R*. *solanacearum* colonization of stem tissues (Bittner et al., 2016).

The same phenomenon of colonization variation also occurs in potato (Ferreira et al., 2017; Sebastià et al., 2021), alfalfa (Turner et al., 2009). In more depth, researchers have studied the structure of plant roots and stems. The results showed that in tomato the xylem vessel were larger in resistant material than in susceptible varieties (Caldwell et al., 2017). This difference may allow larger numbers of *R*. *solanacearum* to colonize without the xylem vessel being completely blocked. In addition, in resistant tomato, the structure of the cell wall and striatal membrane also show differences (Nakaho et al., 2000). The differences in inducible structural defenses and cell walls exhibited by different resistant plants will be elaborated later.

## Induced structural defenses: Tyloses, gels, and callose

**Figure 2:**
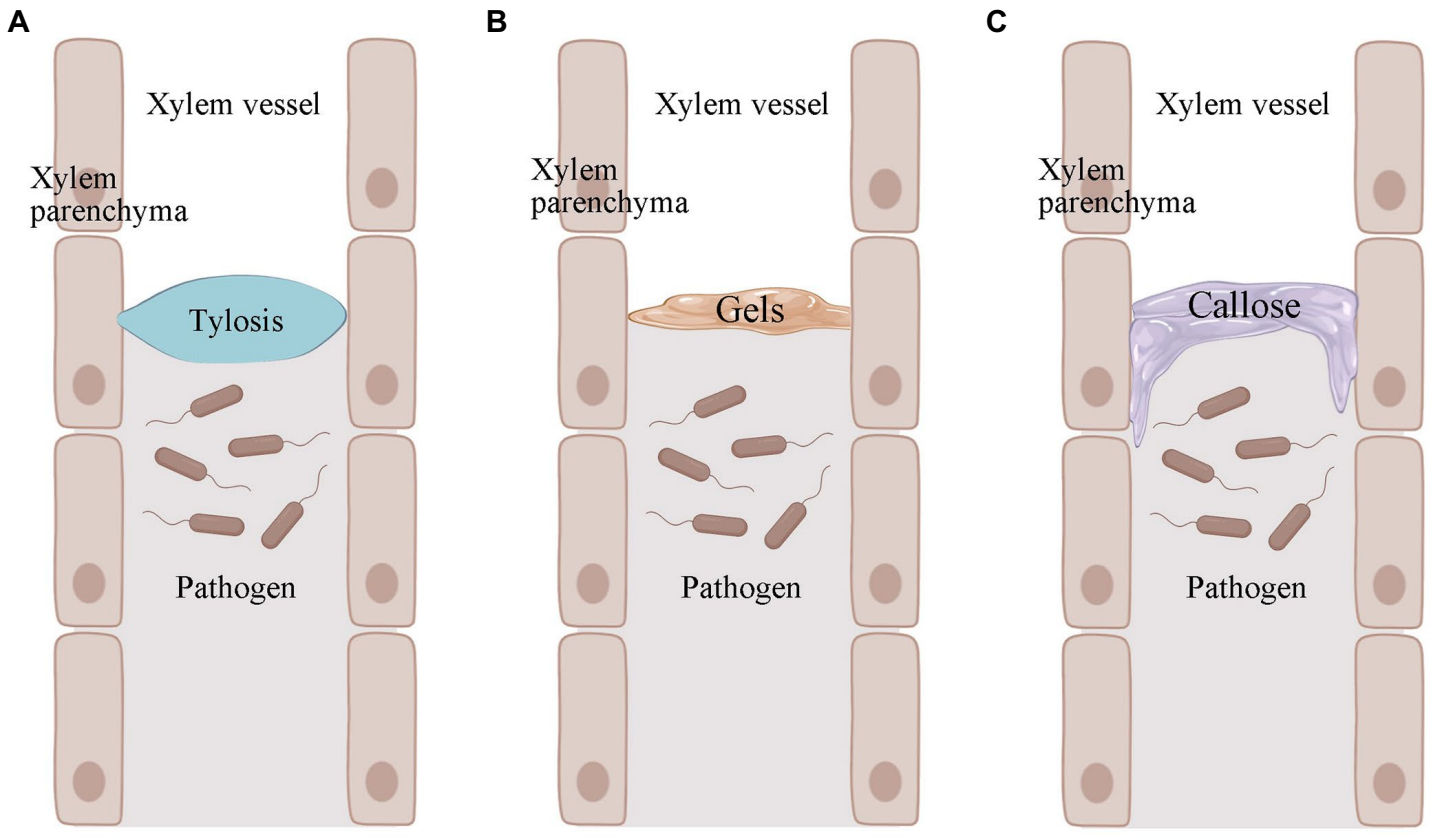
Pathogen-induced production of tyloses, gels, and callose in plants. (**A)** Pathogen-induced plant production of tyloses. (**B)** Pathogen-induced plant production of gels. (**C)** Callose produced by pathogen-induced plants. Images were drawn through BioRender (https://biorender.com/).

Resistant plants cannot prevent the entry of *R*. *solanacearum*, but they can limit the movement of *R*. *solanacearum* (Pruitt et al., 2021). The wide range, number of variants, and regional variation of *R*. *solanacearum* make it difficult to find specific resistance genes that work against all *R*. *solanacearum* variants. Given the uniqueness of *R*. *solanacearum*, it was realized that studying differences in host structural defenses might be a more effective strategy (Kashyap et al., 2020). Among many plants, tomato has been used as a broad model plant to study induced structural defenses against *R*. *solanacearum* (Caldwell et al., 2017). These structural defenses mainly include tyloses, gels, and callose (Figure 2; Kashyap et al., 2020). They can confine the *R*. *solanacearum* within the infected vessel and prevent their further spread (Planas-Marquès et al., 2019).

## Tyloses deposition against *Ralstonia solanacearum*

Tyloses is a structure in which thin-walled cells of a plant grow into the lumen of an adjacent duct and reach into the xylem vessel (Figure 2A; Bonsen and Kučera, 1990). This structure blocks the infected plant vessel, thus preventing further upward spread of *R*. *solanacearum* (Leśniewska et al., 2016; Kashyap et al., 2020). Tyloses formation has been observed at infected sites in both *R*. *solanacearum*-resistant tomato and potato varieties (Grimault et al., 1994; Ferreira et al., 2017). The formation of tyloses was delayed and less concentrated in *R*. *solanacearum*-susceptible tomato. Many vessel that were not colonized by *R*. *solanacearum* were also blocked by the infestation, but pathogen multiplication was not restricted (Grimault et al., 1994). The formation of tyloses was not observed in *R*. *solanacearum*-susceptible tomato varieties when not inoculated with *R*. *solanacearum*, but was present in resistant tomato (Grimault et al., 1994). This pre-formed structural defense may be more timely and effective in the face of *R*. *solanacearum* infestation. In addition to *R*. *solanacearum*, *Fusarium oxysporum* f. sp. *cubense* (VanderMolen et al., 1987), *Ophiognomonia clavigignenti-juglandacearum* (Rioux et al., 2018), *Fusarium oxysporum* f. sp. *vasinfectum* (Shi et al., 1991), *Fusarium oxysporum* f. sp. *melonis* (Seo and Kim, 2017), and other pathogenic bacteria can also induce the production of tyloses in the corresponding plants. In addition, plants can also produce tyloses when subjected to freezing and mechanical damage. This suggests that infestation formation is a stress response of plants in response to biotic and abiotic stresses.

When plants are infested with pathogenic bacteria, secreted effector proteins induce and inhibit the production of related hormones (Landry et al., 2020). In turn, changes in hormone levels can induce or inhibit the formation of the tyloses. The *R*. *solanacearum* effector proteins RipAL, RipR, RipG1, RipG3 and RipAY can induce jasmonic acid (JA) production and inhibit salicylic acid (SA) signaling (Sang et al., 2016; Nakano and Mukaihara, 2018). It has also been shown that JA synergistically interacts with ethylene (ET) to trigger the formation of tyloses. SA and 1-aminocyclopropane-1-carboxylic acid (ACC) have inhibitory and synergistic effects on JA-induced tyloses, respectively (de Nicolai and Rodrigues, 2022). In contrast, the ability of ACC to stimulate tyloses formation is dependent on ET signal transduction. When pruned grape stems were treated with inhibitors of ET biosynthesis and inhibitors of ET action, tyloses formation was delayed or reduced (Sun et al., 2008). Although the molecular mechanisms by which phytohormones regulate tyloses against *R*. *solanacearum* remain unclear, tyloses can play a defensive role as a means for resistant plants to resist *R*. *solanacearum*.

## Gel deposition against *Ralstonia solanacearum*

The formation of tyloses in plants is accompanied by the secretion of gels (Rioux et al., 1998). The main component of the gel is pectin, which also contains some antimicrobial compounds (Clérivet et al., 2000). The gel is usually secreted by xylem thin-walled tissue cells and transported through the striatal membrane into the plant vessel (Figure 2B; Bishop and Cooper, 1984). The gel is an important component of resistance to several wilt diseases. For example, colonization by *F*. *oxysporum* f. sp. *dianthi* causes gel formation in the vascular bundles of carnations (Baayen and Elgersma, 1985). The pea was subjected to *Fusarium oxysporum* f. sp. *matthiolae* infection (Bishop and Cooper, 1984). In addition, gel formation in xylem is one of the characteristics of bacterial wilt-resistant tomato varieties when infested with *R*. *solanacearum* (Kashyap et al., 2020). However, it is worth noting that fungi and bacteria do not necessarily induce the same gel production in plants. There is a correlation between gel formation and season. After pruning, grapes produced mainly gels in winter and tyloses in summer and autumn (Sun et al., 2008). Schmitt and Liese (1992) found that birch and linden stem wound-induced gel secretion was higher in summer and autumn and lower or non-existent in winter. Thus, the association between gel formation and season may be directly related to temperature. Unfortunately, we do not have more evidence for the role of gel deposition in the roots or stems of plants in resisting *R*. *solanacearum*. This may require further studies in the future to provide stronger evidence. However, we cannot deny the role of gels in induced structural defense in plants.

## Callose deposition against *Ralstonia solanacearum*

In the plant duct system, the callose acts as another structural barrier that has been shown to be useful in limiting the horizontal movement of pathogenic bacteria (Figure 2C; Kashyap et al., 2020). When plants are infested with pathogens, the callose is deposited between the plasma membrane and the cell wall. This pathogen-induced callose deposition serves as a chemical and physical defense mechanism to strengthen the plant cell wall and plays an important role in the defense response against invading pathogens (Wang et al., 2021). It has also been suggested that callose may be pre-existing in resistant plants (Ferreira et al., 2017). For example, there was no significant difference in callose in bacterial wilt-resistant potatoe when they were inoculated and not inoculated with *R*. *solanacearum*. Also, this suggests that pre-existing callose deposition in resistant potato may help strengthen the plant cell wall and prevent the spread of *R*. *solanacearum* (Ferreira et al., 2017).

Researchers have demonstrated the role of callose in disease resistance by inhibiting callose synthesis through chemical agents such as 2-deoxy-D-glucose (Wang et al., 2021). For example, when barley mutants were treated with 2-deoxy-D-glucose, early callose formation was reduced and the barley mutants became less resistant to powdery mildew (Bayles et al., 1990). The same approach was observed in the interaction between soybean and soybean mosaic virus (Li et al., 2011) and between tomato and *B*. *cinerea* (Sanmartín et al., 2020). Although the role of callose in disease resistance was demonstrated with the help of chemical reagents, Wang et al. (2021) argued that chemical inhibitors suffer from the problem that inhibitory factors may produce non-specific inhibition of other enzymes. Therefore, studying the disease resistance of callose at the molecular level by means of gene knockout may be a more effective approach. In *Arabidopsis*, the penetrance of *B*. *graminis* in *Arabidopsis* was not significantly altered when the *GSL5* gene (the gene controlling callose production) was knocked out (Jacobs et al., 2003). Although there is evidence that callose as an inducible structural defense is involved in the resistance response to pathogenic bacteria (Ferreira et al., 2017; Kashyap et al., 2020), its role in different species still deserves further investigation. In addition, callose blocks nutrient and water transport while preventing further spread of pathogenic bacteria. Therefore, whether callose is a redundant structural defense in some species needs to be further explored.

## Cell wall involvement in the fight against *Ralstonia solanacearum*

The cell wall is the first barrier of plants against invasion by pathogenic bacteria (Bacete et al., 2018). Plant-*R*. *solanacearum* associated transcriptomic data suggest the involvement of the cell wall in response to *R*. *solanacearum* infestation (Supplementary Table S1). For example, many upregulated genes in resistant tobacco 4,411-3 are involved in cell wall macromolecular metabolic processes and cell wall organization or biogenesis after inoculation with *R*. *solanacearum* (Pan et al., 2021). In peanut, cell wall-related genes showed specific expression differences between resistant and susceptible peanuts (Chen et al., 2014). In pepper, genes associated with xylan biosynthesis and cell wall organization were significantly enriched in response to *R*. *solanacearum* infestation (Hwang et al., 2011). Other plants such as *Arabidopsis* (Zhao et al., 2019), tomato (French et al., 2018), potato (Zuluaga et al., 2015), ginger (Snigdha and Prasath, 2021), and aubergine (Chen et al., 2018) showed a similar situation after inoculation with *R*. *solanacearum*. All of this evidence suggests a role for the cell wall in defense against *R*. *solanacearum* infestation.

To break through cell wall defenses, pathogens often secrete cell wall degrading enzymes including cellulase, pectinase, xylanase, and xyloglucanase to promote infestation (Wanjiru et al., 2002; Lev and Horwitz, 2003; Niture et al., 2006). The plant immune system activates a defense response by sensing cell wall fragments broken down by pathogens (Jones and Dangl, 2006). For example, fibrous dextrins from cellulose induce ROS production and upregulation of PR genes. Oligogalacturonic acid in pectin is sensed by *WAK1* and promotes ROS production. Oligoglucan can lead to callose deposition and increased hormone biosynthesis (Wan et al., 2021). Furthermore, when cell wall integrity (CWI) is compromised, the CWI system senses the state of the cell wall, which activates a defense response (Gigli-Bisceglia et al., 2019; Wan et al., 2021). Several families of plant proteins have been shown to be involved in the detection of CWI damage. For example, the leucine-rich repeat receptor kinase *MIK2* is involved as a regulator of fibrin damage (Van der Does et al., 2017). *THE1*, a member of the *CrRLK1L* protein family, is involved in the response to CWI damage caused by cellulose reduction (Hématy et al., 2007). Individual components of the plant cell wall play different regulatory roles in plant immunity and changes in their composition or structure have been shown to affect plant resistance to pathogenic bacteria (Höfte and Voxeur, 2017; Wan et al., 2021).

Some evidence has shown that components of the plant cell wall such as cellulose, hemicellulose, pectin, lignin, and suberin are involved in defense against pathogens. For example, blockage of the cellulose synthesis pathway can lead to enhanced or reduced plant resistance (Ramírez et al., 2011; Douchkov et al., 2016). When defects in the subunits of CESAs required for secondary cell wall synthesis in *Arabidopsis* lead to resistance to necrotrophic fungi (*Plectosphaerella cucumerina*), gray mold (*Botrytis cinerea*), vascular bacteria (*R*. *solanacearum*), and vascular fungi (*Fusarium oxysporum*; Hernández-Blanco et al., 2007; Escudero et al., 2017). Furthermore, in *Arabidopsis thaliana*, resistance to *Fusarium oxysporum* is enhanced in the presence of defects in *MYB46*, a transcription factor that directly regulates the expression of the *CESA4/7/8* genes (Ramírez et al., 2011). Specific cell wall damage activates different immune responses. However, inhibition of cellulose synthesis does not always lead to increased resistance to disease. For example, transient silencing of cellulose synthase-like D2 (CSLD2) enhances the susceptibility of barley to powdery mildew (Douchkov et al., 2016). When cell wall cellulose synthesis was enhanced, plants showed resistance to the disease. For example, when the transcription factor *OsMYB63*, which promotes the expression of three secondary cell wall-associated cellulose synthase genes, was overexpressed, rice cell walls were thickened and showed increased resistance to *Xanthomonas oryzae* pv. *oryzae* (*Xoo*; Xie et al., 2021). In contrast, when *OsMYB63* was knocked out, the rice cell wall became thinner and showed susceptibility to *Xoo*. In addition, changes in hemicellulose in the cell wall affect plant resistance to pathogenic bacteria (Sampedro et al., 2010; Chowdhury et al., 2017). *Arabidopsis* mutants *det3* and *irx6-1* contain more xylose in their cell walls than the wild type, and these mutants both enhance resistance to *P*. *cucumerina* (Brown et al., 2005; Rogers et al., 2005). In addition, the *Arabidopsis* mutant *xyl1-2* exhibits xyloglucan modifications that also enhance resistance to *P*. *cucumerina* (Sampedro et al., 2010). Resistance to powdery mildew in barley is enhanced when xylan synthesis-related glycosyltransferases are overexpressed (Chowdhury et al., 2017). Acetylation of hemicellulose affects plant resistance to pathogenic bacteria (Wan et al., 2021). This is largely dependent on two protein families (RWA and TBL; Gille et al., 2011; Manabe et al., 2011). In *Arabidopsis*, the mutant *rwa2* lacks acetyltransferase but enhances resistance to *Botrytis cinerea* (Manabe et al., 2011). The *Arabidopsis* mutant *pmr5* downregulated the expression of the *TBL44* gene, resulting in a significant increase in resistance to powdery mildew (Gille et al., 2011). The *Arabidopsis* TBL member *ESK1* encodes an O-acetyltransferase involved in xylan acetylation. Its mutant *esk1* has reduced xylan acetylation but its resistance to *P*. *cucumerina* is enhanced (Escudero et al., 2017).

Once the pathogen has broken through the cuticle of the plant, pectin becomes an important barrier against invasion (Wan et al., 2021). Altered or modified pectin composition can also affect plant resistance to pathogenic bacteria (Bacete et al., 2018). In *Arabidopsis thaliana*, impairment of the pectin biosynthetic pathway diminished resistance of *Arabidopsis* to *Pseudomonas syringae* and *Botrytis cinerea* (Bethke et al., 2016; Zhang et al., 2016). On the other hand, the pectin-rich cell wall of the *Arabidopsis* mutant *pmr5* exhibited susceptibility to *Pseudomonas syringae* and *Peronospora parasitica* (Vogel et al., 2004). However, the methyl esterification and O-acetylation of pectin were lower in *pmr5* compared to the wild type. Pectin modifies itself by methylation or acetylation to increase its complexity (Atmodjo et al., 2013). The methyl esterification of pectin is mainly controlled by pectin methyl esterase (PME) and its activity is regulated by pectin methyl esterase inhibitors (PMEI; Wolf et al., 2009). Interestingly, *pmr5* is resistant to *Erysiphe cichoracearum* and *Erysiphe orontii* (Vogel et al., 2004). Moreover, *pmr5* can also limit the infestation of *Colletotrichum higginsianum* (Engelsdorf et al., 2016). The association between pectin content, methyl esterification, and O-acetylation in plant cell walls and disease resistance is a question worth exploring. In one of our unpublished data, a near-isogenic line of tobacco variety Cuibi-1 (susceptible to *R*. *solanacearum*), KCB-1 (highly resistant to *R*. *solanacearum*), had significantly higher pectin content in the root cell wall than Cuibi-1. The colonization results indicated that KCB-1 had less *R*. *solanacearum* colonization. Therefore, the relationship between pectin and plant bacterial wilt resistance is a direction worth exploring.

Highly methylated pectins show good tolerance to cell wall degrading enzymes secreted by pathogens, thus conferring disease resistance to the plant (Raiola et al., 2011). Studies have demonstrated that overexpression of PMEI enhances plant resistance to pathogens (Lionetti et al., 2007, 2013). For example, *Arabidopsis* showed resistance to powdery mildew and soft rot due to increased pectin methyl esterification when PMEI1 and PMEI2 were overexpressed (Lionetti et al., 2007). *Arabidopsis* resistance to *Pseudomonas syringae* pv. *tomato* was enhanced when CaPMEI1 was overexpressed (An et al., 2008). In addition, kiwifruit PMEI was shown to limit fungal infections caused by *Bipolaris sorokiniana*, *F*. *graminearum*, and *Claviceps purpurea* in wheat (Volpi et al., 2011, 2013). Tobacco mosaic virus symptoms were reduced in tobacco heterologously expressing Kiwi PMEI (Liu et al., 2018). Overexpression of *AtPMEI-2* in *Arabidopsis* significantly reduced its susceptibility to turnip vein-clearing virus (Lionetti et al., 2013). In addition, plants overexpressing PMEI in some *Arabidopsis* strains showed greater susceptibility to virus infection, suggesting a differential effect of pectin methylation on disease resistance (Lionetti et al., 2013). Acetylation of pectin can also enhance plant resistance to pathogenic bacteria. For example, overexpression of aspergillus nidulans acetylase, which reduces pectin acetylation in *Arabidopsis thaliana*, leads to callose deposition and increased resistance to *Botrytis cinerea* (Pogorelko et al., 2013).

Lignin, an important component of the secondary cell wall, often acts as a physical barrier against pathogenic bacteria (Zhang et al., 2021). Lignin acts as a barrier mainly by increasing the mechanical strength of the plant cell wall and improving its resistance to cell wall degrading enzymes released by the pathogen (Hernández-Blanco et al., 2007; Hückelhoven, 2007; Wei et al., 2021). Some transcriptomic data suggest an association between resistance pathways and lignin biosynthesis in *R*. *solanacearum*-resistant tobacco. For example, RNA-Seq data for *R*. *solanacearum*-tobacco show that the benzyl-propane pathway is the main resistance pathway for *R*. *solanacearum* infection. In turn, the phenylpropane pathway is essential for lignin synthesis. Lignin also plays a complex role in the defense against pathogenic bacteria. In a variety of plants, lignin deposition shows enhanced resistance to pathogenic bacteria. In *Arabidopsis*, for example, lignin prevents further proliferation of pathogenic bacteria by accumulating in the leaves (Lee et al., 2019). *MYB15* enhances *Arabidopsis* defense against *Pseudomonas syringae* by regulating the expression levels of G-lignin biosynthesis-related genes (Chezem et al., 2017). In maize, lignin accumulation resulting from inhibition of *ZmCAD* encoded biosynthetic enzymes limits lesion expansion in leaf sheath blight (Li et al., 2019). In addition, loss-of function in rice *Bsr-k1* resulted in increased expression of *OsPAL1-7*, which promoted lignification and broad-spectrum resistance to *Magnaporthe oryzae* and *Xanthomonas oryzae* pv *oryzae* (Zhou et al., 2018). In tomato, one of the differences in resistance between *R*. *solanacearum*-resistant and susceptible tomatoes lies in the assembly of a structural barrier formed by a lignin-corky coating and tyramine-derived hydroxycinnamic acids amides (HCAAs) on the duct system of resistant tomatoes to specifically respond to *R*. *solanacearum* infestation. In contrast, *R*. *solanacearum*-susceptible tomato varieties exhibit degradation of lignin (Kashyap et al., 2021). In addition, significant differences in lignin composition between the susceptible tomato variety Marmande and the resistant tomato variety Hawaii 7,996 suggest that the nature of paravascular lignin may be critical for resistance to *R*. *solanacearum* in resistant plants (Kashyap et al., 2021). There is also evidence that inhibition of the lignin biosynthetic pathway also manifests itself as increased plant resistance.. For example, when the transcription factor *GhMYB4* was overexpressed in cotton, the lignin content of cotton stems was reduced, but its resistance to *Verticillium dahliae* was enhanced (Xiao et al., 2021). In tomato, the lignin biosynthesis gene of the *R*. *solanacearum*-resistant tomato variety LS-89 was upregulated after infection with *R*. *solanacearum* (Ishihara et al., 2012). Plants show different resistance strategies in response to lignin biosynthesis promotion or inhibition. Lignin deposition in the secondary cell wall increases the thickness of the cell wall, thereby enhancing plant resistance. In contrast, when lignin levels are reduced, this leads to changes in cell wall integrity (CWI), which enhances the release of oligogalacturonides (OGs), thereby inducing a deeper immune response in plants (Xiao et al., 2021).

Suberin is a chemically complex heterogeneous polymer (Vishwanath et al., 2014; Andersen et al., 2015) that forms a hydrophobic protective barrier between the plasma membrane and the cell wall (Kashyap et al., 2020). In addition to providing strength to the cell wall, this barrier also prevents water loss and pathogen entry by sealing off the keratinized cell layer. There is considerable variation in the total and relative amount of suberin between developmental stages, tissues, and plant species (Ranathunge and Schreiber, 2011). The metabolome of late leaf spot resistant and susceptible peanut shows that the corky biosynthetic pathway is one of the important pathways of the resistance response (Mahatma et al., 2021). In addition, the induced lignin-corky vascular coating in tomato restricted the colonization of *R*. *solanacearum* in tomato resistant roots (Kashyap et al., 2021). Although there is no further evidence for the role of suberin in defense against *R*. *solanacearum* in other plant-*R*. *solanacearum* interactions, its role as an important component of the cell wall cannot be ignored.

## Plant metabolites involved in the fight against *Ralstonia solanacearum*

Plants infested with pathogens produce a large number of secondary metabolites, some of which are resistant metabolites that disrupt the structure of the pathogen and inhibit its growth and reproduction (Table 1). For example, coumarin inhibits acylhomoserine lactone synthesis, antagonizes quorum sensing (QS) regulatory proteins, and blocks receptor proteins in *R*. *solanacearum* (Qais et al., 2021). Other coumarins, daphnetin, inhibited the production of extracellular polysaccharides (EPS) and biofilm formation in *R*. *solanacearum in vitro* by suppressing gene expression of *xpsR*, *epsE*, *epsB*, and *lexM* (Yang et al., 2021a). 6-Methylcoumarin causes cell elongation, disrupts cell division, and inhibits the expression of *ftsZ*, the gene encoding cytokinin (Yang et al., 2021b). 7-methoxycoumarin inhibits the growth of *R*. *solanacearum* (Yang et al., 2021a) and suppresses its virulence-related genes *epsE*, *hrpG* and *popA* (Han et al., 2021). Hydroxycoumarins can inhibit the expression of *R*. *solanacearum* flagellar genes *fliA* and *flhC*, and disrupt their cell membranes and inhibit biofilm formation (Yang et al., 2016). In addition, the coumarins esculetin, umbelliferone, and others have also been shown to affect *R*. *solanacearum* biofilms (Yang et al., 2016). Other plant resistance metabolites, such as caffeic acid, effectively activate phenylalanine aminolytic enzyme (PAL) and peroxidase (POD) in tobacco and promote the accumulation of lignin and hydroxyproline (Li et al., 2021). Caffeic acid significantly inhibits biofilm formation in *R*. *solanacearum* by suppressing the expression of *lecM* and *epsE* genes (Li et al., 2021).When exogenously applied, caffeic acid significantly reduced and delayed the development of tobacco brucellosis. Methyl gallate inhibited brucellosis by damaging the cell wall structure of the Brucella (Fan et al., 2013). Biochemical analysis showed that methyl gallate could inhibit protein synthesis and succinate dehydrogenase activity in *R*. *solanacearum*. Higher concentrations of methyl gallate can inhibit the respiration of *R*. *solanacearum*, ultimately acting as a fungicide (Fan et al., 2013). In addition, some other metabolites have been shown to inhibit the growth and reproduction of *R*. *solanacearum*, although the mechanism of resistance has not been elucidated (Table 1).

**Table 1:** Role of plant resistance metabolites in resistance to *Ralstonia* *solanacearum*.

Metabolites	Function	References
Daphnetin	Inhibits the production of extracellular polysaccharides (EPS) and biofilm formation in *Ralstonia solanacearum*	Yang et al. (2021b)
6-methylcoumarin	Disturbance of *Ralstonia solanacearum* division	Yang et al. (2021a)
Coumarin	Inhibits QS and biofilm formation of *Ralstonia solanacearum* Inhibits early adhesion and colonization of *Ralstonia solanacearum* in tobacco plants	Qais et al. (2021)
Caffeic acid	Breaks the membrane structure of *Ralstonia solanacearum*	Li et al. (2021)
7-methoxycoumarin	Inhibits the growth of *Ralstonia solanacearum* and disrupts the cell membrane of *Ralstonia solanacearum*	Han et al. (2021)
Esculetin	Inhibition of *Ralstonia solanacearum* biofilm formation	Yang et al. (2016)
Umbelliferone	Inhibition of *Ralstonia solanacearum* biofilm formation	Yang et al. (2016)
Hydroxycoumarins	Disruption of *Ralstonia solanacearum* cell membranes and inhibition of biofilm formation	Yang et al. (2016)
Methyl gallate	Damage to *Ralstonia solanacearum* cell wall	Fan et al. (2013)
Lansiumamide B^*^	–	Li et al. (2014)
Flavonoids^*^	–	Zhao et al. (2011)
Resveratrol	Inhibits early adhesion and colonization of *Ralstonia solanacearum* in tobacco plants. Disrupts *Ralstonia solanacearum* cell membranes. Prevents *Ralstonia solanacearum* swimming and biofilm formation	Chen et al. (2016)
Protocatechualdehyde	Inhibits the growth and biofilm formation of *Ralstonia solanacearum* and disrupts the cell structure and shape of *Ralstonia solanacearum*	Li et al. (2016)
Methanol^*^	–	Murthy (2015)
Ethanol^*^	–	Gaitonde and Ramesh (2016)
Acetone^*^	–	Gaitonde and Ramesh (2016)
Ethyl acetate^*^	–	Gaitonde and Ramesh (2016)
Essential oil^*^	–	Li and Yu (2014)
Ferruginol^*^	–	Matsushita et al. (2006)
Sandaracopimarinol^*^	–	Matsushita et al. (2006)
Liquiritigenin^*^	–	Zhao et al. (2011)
Isoliquiritigenin^*^	–	Zhao et al. (2011)
(3R)-vestitol^*^	–	Zhao et al. (2011)
Protocatechualdehyde^*^	–	Li et al. (2016)
Gallic acid^*^	–	Vu et al. (2013)
4, 6-di-O-galloylarbutin^*^	–	Vu et al. (2013)
2, 6-di-O-galloylarbutin^*^	–	Vu et al. (2013)
2, 4, 6-tri-O-galloyl-glucose^*^	–	Vu et al. (2013)
1, 3, 4, 6-tetra-O-galloyl-β-glucose^*^	–	Vu et al. (2013)
1, 2, 4, 6-tetra-O-galloyl-β-glucose^*^	–	Vu et al. (2013)
1, 2, 3, 6-tetra-O-galloyl-β-glucose^*^	–	Vu et al. (2013)
Eugenol^*^	–	Bai et al. (2016)
Mukaadial^*^	–	Opiyo et al. (2011)
Muzigadial^*^	–	Opiyo et al. (2011)
Polygodial^*^	–	Opiyo et al. (2011)
Ugandensidial^*^	–	Opiyo et al. (2011)
Ugandensolide^*^	–	Opiyo et al. (2011)
Warburganal^*^	–	Opiyo et al. (2011)

The asterisk indicates that the mechanism of resistance of the substance to *R*. *solanacearum* has not been elucidated. Horizontal line represents inhibition of the growth of *R*. *solanacearum*.

In addition to producing resistant metabolites, resistant plants are indirectly involved in the fight against *R*. *solanacearum* by reducing and inhibiting metabolites required by *R*. *solanacearum*. Studies have shown that the plant metabolite L-glutamic is associated with the production of extracellular polysaccharides, cellulase activity, and biofilm formation in *R*. *solanacearum* (Shen et al., 2020). Resistant tomato varieties inhibit the activity of *R*. *solanacearum* by reducing the formation of L-glutamic. It has also been shown that root extracts of *R*. *solanacearum*-susceptible tomato varieties contain various fatty acid derivatives, while the opposite is true in resistant tomato varieties (Zeiss et al., 2018). In tobacco, methyl-alpha-D-glucopyranoside and arabinitol were significantly higher in susceptible tobacco varieties inoculated with *R*. *solanacearum* than in resistant varieties (Yang et al., 2022). Such metabolic markers may be more conducive to the colonization and growth of *R*. *solanacearum*.

## Future prospects

In confrontation with pathogens, plants rely more on induced resistance defense mechanisms to prevent pathogen invasion (De Kesel et al., 2021). Induced resistance in plants limits *R*. *solanacearum* both vertically and horizontally, both physically and chemically, to avoid further spread (Planas-Marquès et al., 2019). Resistant plants that have been produced have shown that the resistance strategy of plants against *R*. *solanacearum* is not to destroy them, but to trap them through their own structure and prevent them from breaking through their cage. The robust structure of the plant itself is therefore extremely important in this process for the control of vascular diseases such as *R*. *solanacearum*. The plant-*R*. *solanacearum* game is a long-term process of confrontation and evolution in which both plants and *R*. *solanacearum* try to use different strategies to outwit each other. It is certainly important to understand the mechanisms of resistance of resistant plants to *R*. *solanacearum* and to deploy strategies in the next step of the breeding process.

## Author contributions

HS collected the relevant literature and wrote the manuscript. AD and WW were involved in the design and direction of the project and revised the content of the manuscript. YL provided important suggestions on manuscript ideas, language revisions, and manuscript revisions. YS provided key guidance in the manuscript writing and revision process and approved the manuscript for submission. All authors were involved in the review and discussion of the manuscript.

## Funding

This work was supported by Key Science and Technology Projects of Sichuan Tobacco Institute (No. SCYC202002), Central Public-Interest Scientific Institution Basal Research Fund (nos. 16102320200002 and 1610232016017), Evaluation of Tobacco Bacterial Wilt Resistance Resources and Study on Resistance Mechanism (no. 110202001024(JY-07)), and Breeding of New Tobacco Strains with Resistance to Bacterial Wilt and Identification of New Resistance Sources (no. 2020530000241008).

## Conflict of interest

The authors declare that the research was conducted in the absence of any commercial or financial relationships that could be construed as a potential conflict of interest.

## Publisher’s note

All claims expressed in this article are solely those of the authors and do not necessarily represent those of their affiliated organizations, or those of the publisher, the editors and the reviewers. Any product that may be evaluated in this article, or claim that may be made by its manufacturer, is not guaranteed or endorsed by the publisher.

## References

[ref1] An S. H. Sohn K. H. Choi H. W. Hwang I. S. Lee S. C. Hwang B. K. (2008). Pepper pectin methylesterase inhibitor protein CaPMEI1 is required for antifungal activity, basal disease resistance and abiotic stress tolerance. Planta 228, 61–78. doi: 10.1007/s00425-008-0719-z, PMID: 18327607 PMC2413075

[ref2] Andersen T. G. Barberon M. Geldner N. (2015). Suberization - the second life of an endodermal cell. Curr. Opin. Plant Biol. 28, 9–15. doi: 10.1016/j.pbi.2015.08.004, PMID: 26343015

[ref3] Atmodjo M. A. Hao Z. Mohnen D. (2013). Evolving views of pectin biosynthesis. Annu. Rev. Plant Biol. 64, 747–779. doi: 10.1146/annurev-arplant-042811-105534, PMID: 23451775

[ref4] Baayen R. P. Elgersma D. M. (1985). Colonization and histopathology of susceptible and resistant carnation cultivars infected with *Fusarium oxysporum* f. sp. *dianthi*. Neth. J. Plant Pathol. 91, 119–135. doi: 10.1007/bf01976386

[ref5] Bacete L. Mélida H. Miedes E. Molina A. (2018). Plant cell wall-mediated immunity: cell wall changes trigger disease resistance responses. Plant J. 93, 614–636. doi: 10.1111/tpj.13807, PMID: 29266460

[ref6] Bae C. Han S. W. Song Y.-R. Kim B.-Y. Lee H.-J. Lee J.-M.. (2015). Infection processes of xylem-colonizing pathogenic bacteria: possible explanations for the scarcity of qualitative disease resistance genes against them in crops. Theor. Appl. Genet. 128, 1219–1229. doi: 10.1007/s00122-015-2521-1, PMID: 25917599

[ref7] Bai W. Kong F. Lin Y. Zhang C. (2016). Extract of *Syringa oblata*: a new biocontrol agent against tobacco bacterial wilt caused by *Ralstonia solanacearum*. Pestic. Biochem. Physiol. 134, 79–83. doi: 10.1016/j.pestbp.2016.04.002, PMID: 27914543

[ref8] Bayles C. J. Ghemawat M. S. Aist J. R. (1990). Inhibition by 2-deoxy-D-glucose of callose formation, papilla deposition, and resistance to powdery mildew in an ml-o barley mutant. Physiol. Mol. Plant Pathol. 36, 63–72. doi: 10.1016/0885-5765(90)90092-c

[ref9] Bethke G. Thao A. Xiong G. Li B. Soltis N. E. Hatsugai N.. (2016). Pectin biosynthesis is critical for cell wall integrity and immunity in *Arabidopsis thaliana*. Plant Cell 28, 537–556. doi: 10.1105/tpc.15.00404, PMID: 26813622 PMC4790862

[ref10] Bishop C. D. Cooper R. M. (1984). Ultrastructure of vascular colonization by fungal wilt pathogens. II: Invasion of resistant cultivars. Physiol. Plant Pathol. 24, 277–289. doi: 10.1016/0048-4059(84)90002-x

[ref11] Bittner R. J. Arellano C. Mila A. L. (2016). Effect of temperature and resistance of tobacco cultivars to the progression of bacterial wilt, caused by *Ralstonia solanacearum*. Plant Soil 408, 299–310. doi: 10.1007/s11104-016-2938-6

[ref12] Bonsen K. J. M. Kučera L. J. (1990). Vessel occlusions in plants: morphological, functional and evolutionary aspects. IAWA J. 11, 393–399. doi: 10.1163/22941932-90000528

[ref13] Brown D. M. Zeef L. A. H. Ellis J. Goodacre R. Turner S. R. (2005). Identification of novel genes in *Arabidopsis* involved in secondary cell wall formation using expression profiling and reverse genetics. Plant Cell 17, 2281–2295. doi: 10.1105/tpc.105.031542, PMID: 15980264 PMC1182489

[ref14] Caldwell D. Kim B.-S. Iyer-Pascuzzi A. S. (2017). *Ralstonia solanacearum* differentially colonizes roots of resistant and susceptible tomato plants. Phytopathology 107, 528–536. doi: 10.1094/phyto-09-16-0353-r, PMID: 28112595

[ref15] Chen J. Yu Y. Li S. Ding W. (2016). Resveratrol and coumarin: novel agricultural antibacterial agent against *Ralstonia solanacearum in vitro* and *in vivo*. Molecules 21:1501. doi: 10.3390/molecules21111501, PMID: 27834875 PMC6273507

[ref16] Chen N. Yu B. Dong R. Lei J. Chen C. Cao B. (2018). RNA-Seq-derived identification of differential transcription in the eggplant (*Solanum melongena*) following inoculation with bacterial wilt. Gene 644, 137–147. doi: 10.1016/j.gene.2017.11.003, PMID: 29104166

[ref17] Chen Y. Ren X. Zhou X. Huang L. Yan L. Lei Y.. (2014). Dynamics in the resistant and susceptible peanut (*Arachis hypogaea* L.) root transcriptome on infection with the *Ralstonia solanacearum*. BMC Genomics 15:1078. doi: 10.1186/1471-2164-15-1078, PMID: 25481772 PMC4300042

[ref18] Chezem W. R. Memon A. Li F.-S. Weng J.-K. Clay N. K. (2017). SG2-type R2R3-MYB transcription factor MYB15 controls defense-induced lignification and basal immunity in *Arabidopsis*. Plant Cell 29, 1907–1926. doi: 10.1105/tpc.16.00954, PMID: 28733420 PMC5590497

[ref19] Chiang Y.-H. Coaker G. (2015). Effector triggered immunity: NLR immune perception and downstream defense responses. Arabidopsis Book 13:e0183. doi: 10.1199/tab.0183

[ref20] Chowdhury J. Lück S. Rajaraman J. Douchkov D. Shirley N. J. Schwerdt J. G.. (2017). Altered expression of genes implicated in xylan biosynthesis affects penetration resistance against powdery mildew. Front. Plant Sci. 8:445. doi: 10.3389/fpls.2017.00445, PMID: 28408913 PMC5374208

[ref21] Clérivet A. Déon V. Alami I. Lopez F. Geiger J.-P. Nicole M. (2000). Tyloses and gels associated with cellulose accumulation in vessels are responses of plane tree seedlings (Platanus × acerifolia) to the vascular fungus *Ceratocystis fimbriata* f. sp *platani*. Trees 15, 25–31. doi: 10.1007/s004680000063

[ref22] Coll N. S. Valls M. (2013). Current knowledge on the *Ralstonia solanacearum* type III secretion system. Microb. Biotechnol. 6, 614–620. doi: 10.1111/1751-7915.12056, PMID: 23617636 PMC3815929

[ref23] Cui H. Tsuda K. Parker J. E. (2015). Effector-triggered immunity: from pathogen perception to robust defense. Annu. Rev. Plant Biol. 66, 487–511. doi: 10.1146/annurev-arplant-050213-040012, PMID: 25494461

[ref24] De Kesel J. Conrath U. Flors V. Luna E. Mageroy M. H. Mauch-Mani B.. (2021). The induced resistance lexicon: do’s and don’ts. Trends Plant Sci. 26, 685–691. doi: 10.1016/j.tplants.2021.01.001, PMID: 33531282

[ref25] de Nicolai J. Rodrigues T. M. (2022). Cell wall thickenings and tylosoid: developmental morphology reveals novelties for secretory canals in *Protium ovatum* (*Burseraceae*). J. Plant Res. 135, 247–257. doi: 10.1007/s10265-021-01365-6, PMID: 34984559

[ref26] Douchkov D. Lueck S. Hensel G. Kumlehn J. Rajaraman J. Johrde A.. (2016). The barley (*Hordeum vulgare*) cellulose synthase-like D2 gene (*HvCslD2*) mediates penetration resistance to host-adapted and nonhost isolates of the powdery mildew fungus. New Phytol. 212, 421–433. doi: 10.1111/nph.14065, PMID: 27352228

[ref27] Engelsdorf T. Will C. Hofmann J. Schmitt C. Merritt B. B. Rieger L.. (2016). Cell wall composition and penetration resistance against the fungal pathogen *Colletotrichum higginsianumare* affected by impaired starch turnover in *Arabidopsis* mutants. EXBOTJ 68, 701–713. doi: 10.1093/jxb/erw434, PMID: 28204541 PMC5441917

[ref28] Escudero V. Jordá L. Sopeña-Torres S. Mélida H. Miedes E. Muñoz-Barrios A.. (2017). Alteration of cell wall xylan acetylation triggers defense responses that counterbalance the immune deficiencies of plants impaired in the β-subunit of the heterotrimeric G-protein. Plant J. 92, 386–399. doi: 10.1111/tpj.13660, PMID: 28792629 PMC5641240

[ref29] Fan W.-W. Yuan G.-Q. Li Q.-Q. Lin W. (2013). Antibacterial mechanisms of methyl gallate against *Ralstonia solanacearum*. Austr. Plant Pathol. 43, 1–7. doi: 10.1007/s13313-013-0234-y

[ref30] Ferreira V. Pianzzola M. J. Vilaró F. L. Galván G. A. Tondo M. L. Rodriguez M. V.. (2017). Interspecific potato breeding lines display differential colonization patterns and induced defense responses after *Ralstonia solanacearum* infection. Front. Plant Sci. 8:1424. doi: 10.3389/fpls.2017.01424, PMID: 28894453 PMC5581342

[ref31] French E. Kim B.-S. Rivera-Zuluaga K. Iyer-Pascuzzi A. S. (2018). Whole root transcriptomic analysis suggests a role for auxin pathways in resistance to *Ralstonia solanacearum* in tomato. MPMI 31, 432–444. doi: 10.1094/mpmi-08-17-0209-r, PMID: 29153016

[ref32] Gaitonde S. S. Ramesh R. (2016). Screening plant provessel for *Ralstonia solanacearum* inhibition and characterization of antibacterial compounds in *Garcinia indica* and *Tamarindus indica*. Proc. Natl. Acad. Sci. India Sect. B Biol. Sci. 88, 265–276. doi: 10.1007/s40011-016-0755-6

[ref33] Gigli-Bisceglia N. Engelsdorf T. Hamann T. (2019). Plant cell wall integrity maintenance in model plants and crop species-relevant cell wall components and underlying guiding principles. Cell. Mol. Life Sci. 77, 2049–2077. doi: 10.1007/s00018-019-03388-8, PMID: 31781810 PMC7256069

[ref34] Gille S. de Souza A. Xiong G. Benz M. Cheng K. Schultink A.. (2011). O-acetylation of *Arabidopsis* hemicellulose xyloglucan requires *AXY4* or *AXY4L*, proteins with a TBL and DUF231 domain. Plant Cell 23, 4041–4053. doi: 10.1105/tpc.111.091728, PMID: 22086088 PMC3246330

[ref35] Grimault V. Gélie B. Lemattre M. Prior P. Schmit J. (1994). Comparative histology of resistant and susceptible tomato cultivars infected by *Pseudomonas solanacearum*. Physiol. Mol. Plant Pathol. 44, 105–123. doi: 10.1016/s0885-5765(05)80105-5

[ref36] Grimault V. Prior P. (1993). Bacterial wilt resistance in tomato associated with tolerance of vascular tissues to *pseudomonas solanacearum*. Plant Pathol. 42, 589–594. doi: 10.1111/j.1365-3059.1993.tb01539.x

[ref37] Han S. Yang L. Wang Y. Ran Y. Li S. Ding W. (2021). Preliminary studies on the antibacterial mechanism of a new plant-derived compound, 7-Methoxycoumarin, against *Ralstonia solanacearum*. Front. Microbiol. 12:7911. doi: 10.3389/fmicb.2021.697911, PMID: 34421853 PMC8377673

[ref38] Hématy K. Sado P.-E. Van Tuinen A. Rochange S. Desnos T. Balzergue S.. (2007). A receptor-like kinase mediates the response of *Arabidopsis* cells to the inhibition of cellulose synthesis. Curr. Biol. 17, 922–931. doi: 10.1016/j.cub.2007.05.018, PMID: 17540573

[ref39] Hernández-Blanco C. Feng D. X. Hu J. Sánchez-Vallet A. Deslandes L. Llorente F.. (2007). Impairment of cellulose synthases required for *Arabidopsis* secondary cell wall formation enhances disease resistance. Plant Cell 19, 890–903. doi: 10.1105/tpc.106.048058, PMID: 17351116 PMC1867366

[ref40] Höfte H. Voxeur A. (2017). Plant cell walls. Curr. Biol. 27, R865–R870. doi: 10.1016/j.cub.2017.05.025 28898654

[ref41] Hückelhoven R. (2007). Cell wall–associated mechanisms of disease resistance and susceptibility. Annu. Rev. Phytopathol. 45, 101–127. doi: 10.1146/annurev.phyto.45.062806.094325, PMID: 17352660

[ref42] Hwang J. Choi Y. Kang J. Kim S. Cho M. Mihalte L.. (2011). Microarray analysis of the transcriptome for bacterial wilt resistance in pepper (*Capsicum annuum* L.). Not. Bot. Hort. Agrobot. Cluj. 39:49. doi: 10.15835/nbha3926820

[ref43] Ishihara T. Mitsuhara I. Takahashi H. Nakaho K. (2012). Transcriptome analysis of quantitative resistance-specific response upon *Ralstonia solanacearum* infection in tomato. PLoS One 7:e46763. doi: 10.1371/journal.pone.0046763, PMID: 23071630 PMC3465262

[ref44] Jacobs A. K. Lipka V. Burton R. A. Panstruga R. Strizhov N. Schulze-Lefert P.. (2003). An *Arabidopsis* callose synthase, *GSL5*, is required for wound and papillary callose formation. Plant Cell 15, 2503–2513. doi: 10.1105/tpc.016097, PMID: 14555698 PMC280557

[ref45] Jones J. D. G. Dangl J. L. (2006). The plant immune system. Nature 444, 323–329. doi: 10.1038/nature05286 17108957

[ref46] Kashyap A. Capellades M. Zhang W. Srinivasan S. Laromaine A. Serra O.. (2021). Induced ligno-suberin vascular coating and tyramine-derived hydroxycinnamic acid amides restrict *Ralstonia solanacearum* colonization in resistant tomato roots. New Phytol. 234, 1411–1429. doi: 10.1101/2021.06.15.448549, 35152435

[ref47] Kashyap A. Planas-Marquès M. Capellades M. Valls M. Coll N. S. (2020). Blocking intruders: inducible physico-chemical barriers against plant vascular wilt pathogens. J. Exp. Bot. 72, 184–198. doi: 10.1093/jxb/eraa444, PMID: 32976552 PMC7853604

[ref48] Landry D. González-Fuente M. Deslandes L. Peeters N. (2020). The large, diverse, and robust arsenal of *Ralstonia solanacearum* type III effectors and their in planta functions. Mol. Plant Pathol. 21, 1377–1388. doi: 10.1111/mpp.12977, PMID: 32770627 PMC7488467

[ref49] Lee M. Jeon H. S. Kim S. H. Chung J. H. Roppolo D. Lee H.. (2019). Lignin-based barrier restricts pathogens to the infection site and confers resistance in plants. EMBO J. 38:e101948. doi: 10.15252/embj.2019101948, PMID: 31559647 PMC6885736

[ref50] Leśniewska J. Öhman D. Krzesłowska M. Kushwah S. Barciszewska-Pacak M. Kleczkowski L. A.. (2016). Defense responses in aspen with altered pectin methylesterase activity reveal the hormonal inducers of tyloses. Plant Physiol. 173, 1409–1419. doi: 10.1104/pp.16.01443, PMID: 27923986 PMC5291032

[ref51] Lev S. Horwitz B. A. (2003). A mitogen-activated protein kinase pathway modulates the expression of two cellulase genes in cochliobolus heterostrophus during plant infection. Plant Cell 15, 835–844. doi: 10.1105/tpc.010546, PMID: 12671080 PMC152332

[ref52] Li C.-M. Yu J.-P. (2014). Chemical composition, antimicrobial activity and mechanism of action of essential oil from the leaves of *Macleaya Cordata* (Willd.) R. Br. J. Food Saf. 35, 227–236. doi: 10.1111/jfs.12175

[ref53] Li L. Feng X. Tang M. Hao W. Han Y. Zhang G.. (2014). Antibacterial activity of Lansiumamide B to tobacco bacterial wilt (*Ralstonia solanacearum*). Microbiol. Res. 169, 522–526. doi: 10.1016/j.micres.2013.12.003, PMID: 24512921

[ref54] Li N. Lin B. Wang H. Li X. Yang F. Ding X.. (2019). Natural variation in *ZmFBL41* confers banded leaf and sheath blight resistance in maize. Nat. Genet. 51, 1540–1548. doi: 10.1038/s41588-019-0503-y, PMID: 31570888

[ref55] Li S. Pi J. Zhu H. Yang L. Zhang X. Ding W. (2021). Caffeic acid in tobacco root exudate defends tobacco plants from infection by *Ralstonia solanacearum*. Front. Plant Sci. 12:586. doi: 10.3389/fpls.2021.690586, PMID: 34456935 PMC8387680

[ref56] Li S. Yu Y. Chen J. Guo B. Yang L. Ding W. (2016). Evaluation of the antibacterial effects and mechanism of action of protocatechualdehyde against *Ralstonia solanacearum*. Molecules 21:754. doi: 10.3390/molecules21060754, PMID: 27294898 PMC6274444

[ref57] Li W. Zhao Y. Liu C. Yao G. Wu S. Hou C.. (2011). Callose deposition at plasmodesmata is a critical factor in restricting the cell-to-cell movement of soybean mosaic virus. Plant Cell Rep. 31, 905–916. doi: 10.1007/s00299-011-1211-y, PMID: 22200865

[ref58] Lionetti V. Raiola A. Camardella L. Giovane A. Obel N. Pauly M.. (2007). Overexpression of pectin methylesterase inhibitors in *Arabidopsis* restricts fungal infection by *Botrytis cinerea*. Plant Physiol. 143, 1871–1880. doi: 10.1104/pp.106.090803, PMID: 17277091 PMC1851811

[ref59] Lionetti V. Raiola A. Cervone F. Bellincampi D. (2013). Transgenic expression of pectin methylesterase inhibitors limits tobamovirus spread in tobacco and *Arabidopsis*. Mol. Plant Pathol. 15, 265–274. doi: 10.1111/mpp.12090, PMID: 24127644 PMC6638747

[ref60] Liu N. Sun Y. Pei Y. Zhang X. Wang P. Li X.. (2018). A pectin methylesterase inhibitor enhances resistance to verticillium wilt. Plant Physiol. 176, 2202–2220. doi: 10.1104/pp.17.01399, PMID: 29363564 PMC5841709

[ref61] Lowe-Power T. M. Khokhani D. Allen C. (2018). How *Ralstonia solanacearum* exploits and thrives in the flowing plant xylem environment. Trends Microbiol. 26, 929–942. doi: 10.1016/j.tim.2018.06.002, PMID: 29941188

[ref62] Mahatma M. K. Thawait L. K. Jadon K. S. Thirumalaisamy P. P. Bishi S. K. Rathod K. J.. (2021). Metabolic profiling for dissection of late leaf spot disease resistance mechanism in groundnut. Physiol. Mol. Biol. Plants 27, 1027–1041. doi: 10.1007/s12298-021-00985-5, PMID: 34108825 PMC8140181

[ref63] Manabe Y. Nafisi M. Verhertbruggen Y. Orfila C. Gille S. Rautengarten C.. (2011). Loss-of-function mutation of reduced wall acetylation2 in *Arabidopsis* leads to reduced cell wall acetylation and increased resistance to *Botrytis cinerea*. Plant Physiol. 155, 1068–1078. doi: 10.1104/pp.110.168989, PMID: 21212300 PMC3046569

[ref64] Matsushita Y. Hwang Y.-H. Sugamoto K. Matsui T. (2006). Antimicrobial activity of heartwood components of sugi (*Cryptomeria japonica*) against several fungi and bacteria. J. Wood Sci. 52, 552–556. doi: 10.1007/s10086-005-0793-9

[ref65] Murthy N. (2015). Antibacterial activity of curcuma longa (turmeric) plant extracts against bacterial wilt of tomato caused by *Ralstonia solanacearum*. Int. J. Sci. Res. 4, 2136–2141.

[ref66] Nakaho K. Hibino H. Miyagawa H. (2000). Possible mechanisms limiting movement of *Ralstonia solanacearum* in resistant tomato tissues. J. Phytopathol. 148, 181–190. doi: 10.1046/j.1439-0434.2000.00476.x

[ref67] Nakaho K. Inoue H. Takayama T. Miyagawa H. (2004). Distribution and multiplication of *Ralstonia solanacearum* in tomato plants with resistance derived from different origins. J. Gen. Plant Pathol. 70, 115–119. doi: 10.1007/s10327-003-0097-0

[ref68] Nakano M. Ichinose Y. Mukaihara T. (2020). *Ralstonia solanacearum* type III effector *RipAC* targets *SGT1* to suppress effector-triggered immunity. Plant Cell Physiol. 61, 2067–2076. doi: 10.1093/pcp/pcaa122, PMID: 32991707

[ref69] Nakano M. Mukaihara T. (2018). *Ralstonia solanacearum* type III effector *RipAL* targets chloroplasts and induces jasmonic acid production to suppress salicylic acid-mediated defense responses in plants. Plant Cell Physiol. 59, 2576–2589. doi: 10.1093/pcp/pcy177, PMID: 30165674

[ref70] Ngou B. P. M. Ahn H.-K. Ding P. Jones J. D. G. (2021). Mutual potentiation of plant immunity by cell-surface and intracellular receptors. Nature 592, 110–115. doi: 10.1038/s41586-021-03315-7, PMID: 33692545

[ref71] Niture S. K. Kumar A. R. Pant A. (2006). Role of glucose in production and repression of polygalacturonase and pectate lyase from phytopathogenic fungus fusarium moniliforme NCIM 1276. World J. Microbiol. Biotechnol. 22, 893–899. doi: 10.1007/s11274-006-9119-3

[ref72] Opiyo S. A. Manguro L. O. A. Okinda-Owuor P. Ateka E. M. Lemmen P. (2011). 7α-Acetylugandensolide and antimicrobial properties of Warburgia ugandensis extracts and isolates against sweet potato pathogens. Phytochem. Lett. 4, 161–165. doi: 10.1016/j.phytol.2011.02.007

[ref73] Pan X. Chen J. Yang A. Yuan Q. Zhao W. Xu T.. (2021). Comparative transcriptome profiling reveals defense-related genes against *Ralstonia solanacearum* infection in tobacco. Front. Plant Sci. 12:7882. doi: 10.3389/fpls.2021.767882.7, PMID: 34970284 PMC8712766

[ref74] Panstruga R. Parker J. E. Schulze-Lefert P. (2009). SnapShot: plant immune response pathways. Cells 136, 978.e1–978.e3. doi: 10.1016/j.cell.2009.02.020, PMID: 19269372

[ref75] Peeters N. Guidot A. Vailleau F. Valls M. (2013). *Ralstonia solanacearum*, a widespread bacterial plant pathogen in the post-genomic era. Mol. Plant Pathol. 14, 651–662. doi: 10.1111/mpp.12038, PMID: 23718203 PMC6638647

[ref76] Planas-Marquès M. Kressin J. P. Kashyap A. Panthee D. R. Louws F. J. Coll N. S.. (2019). Four bottlenecks restrict colonization and invasion by the pathogen *Ralstonia solanacearum* in resistant tomato. J. Exp. Bot. 71, 2157–2171. doi: 10.1093/jxb/erz562, PMID: 32211785 PMC7242079

[ref77] Pogorelko G. Lionetti V. Fursova O. Sundaram R. M. Qi M. Whitham S. A.. (2013). *Arabidopsis* and brachypodium distachyon transgenic plants expressing aspergillus nidulans acetylesterases have decreased degree of polysaccharide acetylation and increased resistance to pathogens. Plant Physiol. 162, 9–23. doi: 10.1104/pp.113.214460, PMID: 23463782 PMC3641233

[ref78] Pruitt R. N. Gust A. A. Nürnberger T. (2021). Plant immunity unified, Plant immunity unified. Nat. Plants 7, 382–383. doi: 10.1038/s41477-021-00903-3 33785867

[ref79] Qais F. A. Khan M. S. Ahmad I. Husain F. M. Khan R. A. Hassan I.. (2021). Coumarin exhibits broad-spectrum antibiofilm and antiquorum sensing activity against gram-negative bacteria: *in vitro* and *in silico* investigation. ACS Omega 6, 18823–18835. doi: 10.1021/acsomega.1c02046, PMID: 34337222 PMC8320077

[ref80] Raiola A. Lionetti V. Elmaghraby I. Immerzeel P. Mellerowicz E. J. Salvi G.. (2011). Pectin methylesterase is induced in *Arabidopsis* upon infection and is necessary for a successful colonization by necrotrophic pathogens. MPMI 24, 432–440. doi: 10.1094/mpmi-07-10-0157, PMID: 21171891

[ref81] Ramírez V. Agorio A. Coego A. García-Andrade J. Hernández M. J. Balaguer B.. (2011). *MYB46* modulates disease susceptibility to Botrytis cinerea in *Arabidopsis*. Plant Physiol. 155, 1920–1935. doi: 10.1104/pp.110.171843, PMID: 21282403 PMC3091096

[ref82] Ranathunge K. Schreiber L. (2011). Water and solute permeabilities of *Arabidopsis* roots in relation to the amount and composition of aliphatic suberin. J. Exp. Bot. 62, 1961–1974. doi: 10.1093/jxb/erq389, PMID: 21421706 PMC3060681

[ref83] Rioux D. Blais M. Nadeau-Thibodeau N. Lagacé M. DesRochers P. Klimaszewska K.. (2018). First extensive microscopic study of butternut defense mechanisms following inoculation with the canker pathogen *Ophiognomonia clavigignenti-juglandacearum* reveals compartmentalization of tissue damage. Phytopathology 108, 1237–1252. doi: 10.1094/phyto-03-18-0076-r, PMID: 29749798

[ref84] Rioux D. Nicole M. Simard M. Ouellette G. B. (1998). Immunocytochemical evidence that secretion of pectin occurs during gel (gum) and tylosis formation in trees. Phytopathology 88, 494–505. doi: 10.1094/phyto.1998.88.6.494, PMID: 18944900

[ref85] Rogers L. A. Dubos C. Surman C. Willment J. Cullis I. F. Mansfield S. D.. (2005). Comparison of lignin deposition in three ectopic lignification mutants. New Phytol. 168, 123–140. doi: 10.1111/j.1469-8137.2005.01496.x, PMID: 16159327

[ref86] Rufián J. S. Lucía A. Rueda-Blanco J. Zumaquero A. Guevara C. M. Ortiz-Martín I.. (2018). Suppression of *HopZ* effector-triggered plant immunity in a natural pathosystem. Front. Plant Sci. 9:977. doi: 10.3389/fpls.2018.00977, PMID: 30154802 PMC6103241

[ref87] Sampedro J. Pardo B. Gianzo C. Guitián E. Revilla G. Zarra I. (2010). Lack of α-xylosidase activity in *Arabidopsis* alters xyloglucan composition and results in growth defects. Plant Physiol. 154, 1105–1115. doi: 10.1104/pp.110.163212, PMID: 20801759 PMC2971592

[ref88] Sang Y. Wang Y. Ni H. Cazalé A.-C. She Y.-M. Peeters N.. (2016). The *Ralstonia solanacearum* type III effector *RipAY* targets plant redox regulators to suppress immune responses. Mol. Plant Pathol. 19, 129–142. doi: 10.1111/mpp.12504, PMID: 27768829 PMC6638004

[ref89] Sanmartín N. Pastor V. Pastor-Fernández J. Flors V. Pozo M. J. Sánchez-Bel P. (2020). Role and mechanisms of callose priming in mycorrhiza-induced resistance. J. Exp. Bot. 71, 2769–2781. doi: 10.1093/jxb/eraa030, PMID: 31985797 PMC7210776

[ref90] Schmitt U. Liese W. (1992). Seasonal influences on early wound reactions in *Betula* and *Tilia*. Wood Sci. Technol. 26:9245. doi: 10.1007/bf00229245

[ref91] Sebastià P. de Pedro-Jové R. Daubech B. Kashyap A. Coll N. S. Valls M. (2021). The bacterial wilt reservoir host *Solanum dulcamara* shows resistance to *Ralstonia solanacearum* infection. Front. Plant Sci. 12:5708. doi: 10.3389/fpls.2021.755708, PMID: 34868145 PMC8636001

[ref92] Seo Y. Kim Y. H. (2017). Pathological interrelations of soil-borne diseases in cucurbits caused by fusarium species and meloidogyne incognita. Plant Pathol. J. 33, 410–423. doi: 10.5423/ppj.oa.04.2017.0088, PMID: 28811758 PMC5538445

[ref93] Shaban M. Miao Y. Ullah A. Khan A. Q. Menghwar H. Khan A. H.. (2018). Physiological and molecular mechanism of defense in cotton against *Verticillium dahliae*. Plant Physiol. Biochem. 125, 193–204. doi: 10.1016/j.plaphy.2018.02.011, PMID: 29462745

[ref94] Shen F. Yin W. Song S. Zhang Z. Ye P. Zhang Y.. (2020). *Ralstonia solanacearum* promotes pathogenicity by utilizing L-glutamic acid from host plants. Mol. Plant Pathol. 21, 1099–1110. doi: 10.1111/mpp.12963, PMID: 32599676 PMC7368120

[ref95] Shi J. Mueller W. C. Beckman C. H. (1991). Ultrastructural responses of vessel contact cells in cotton plants resistant or susceptible to infection by *fusarium oxysporum* f. sp. *vasinfectum*. Physiol. Mol. Plant Pathol. 38, 211–222. doi: 10.1016/s0885-5765(05)80125-0

[ref96] Snigdha M. Prasath D. (2021). Transcriptomic analysis to reveal the differentially expressed miRNA targets and their miRNAs in response to *Ralstonia solanacearum* in ginger species. BMC Plant Biol. 21:355. doi: 10.1186/s12870-021-03108-0, PMID: 34325661 PMC8323298

[ref97] Sun Q. Rost T. L. Matthews M. A. (2008). Wound-induced vascular occlusions in Vitis vinifera (Vitaceae): Tyloses in summer and gels in winter1. Am. J. Bot. 95, 1498–1505. doi: 10.3732/ajb.0800061, PMID: 21628157

[ref98] Turner M. Jauneau A. Genin S. Tavella M.-J. Vailleau F. Gentzbittel L.. (2009). Dissection of bacterial wilt on medicagotruncatula revealed two type III secretion system effectors acting on root infection process and disease development. Plant Physiol. 150, 1713–1722. doi: 10.1104/pp.109.141523, PMID: 19493968 PMC2719136

[ref99] Van der Does D. Boutrot F. Engelsdorf T. Rhodes J. McKenna J. F. Vernhettes S.. (2017). The *Arabidopsis* leucine-rich repeat receptor kinase MIK2/LRR-KISS connects cell wall integrity sensing, root growth and response to abiotic and biotic stresses. PLoS Genet. 13:e1006832. doi: 10.1371/journal.pgen.1006832, PMID: 28604776 PMC5484538

[ref100] VanderMolen G. E. Beckman C. H. Rodehorst E. (1987). The ultrastructure of tylose formation in resistant banana following inoculation with *fusarium oxysporum* f. sp. *cubense*. Physiol. Mol. Plant Pathol. 31, 185–200. doi: 10.1016/0885-5765(87)90063-4

[ref101] Vishwanath S. J. Delude C. Domergue F. Rowland O. (2014). Suberin: biosynthesis, regulation, and polymer assembly of a protective extracellular barrier. Plant Cell Rep. 34, 573–586. doi: 10.1007/s00299-014-1727-z, PMID: 25504271

[ref102] Vogel J. P. Raab T. K. Somerville C. R. Somerville S. C. (2004). Mutations in PMR5 result in powdery mildew resistance and altered cell wall composition. Plant J. 40, 968–978. doi: 10.1111/j.1365-313x.2004.02264.x, PMID: 15584961

[ref103] Volpi C. Janni M. Lionetti V. Bellincampi D. Favaron F. D’Ovidio R. (2011). The ectopic expression of a pectin methyl esterase inhibitor increases pectin methyl esterification and limits fungal diseases in wheat. MPMI 24, 1012–1019. doi: 10.1094/mpmi-01-11-0021, PMID: 21585271

[ref104] Volpi C. Raiola A. Janni M. Gordon A. O’Sullivan D. M. Favaron F.. (2013). *Claviceps purpurea* expressing polygalacturonases escaping PGIP inhibition fully infects PvPGIP2 wheat transgenic plants but its infection is delayed in wheat transgenic plants with increased level of pectin methyl esterification. Plant Physiol. Biochem. 73, 294–301. doi: 10.1016/j.plaphy.2013.10.011, PMID: 24184449

[ref105] Vu T. T. Kim J.-C. Choi Y. H. Choi G. J. Jang K. S. Choi T. H.. (2013). Effect of gallotannins derived from *sedum takesimense* on tomato bacterial wilt. Plant Dis. 97, 1593–1598. doi: 10.1094/pdis-04-13-0350-re, PMID: 30716836

[ref106] Wan J. He M. Hou Q. Zou L. Yang Y. Wei Y.. (2021). Cell wall associated immunity in plants. Stress Biol. 1:4154. doi: 10.1007/s44154-021-00003-4 PMC10429498 37676546

[ref107] Wang Y. Li X. Fan B. Zhu C. Chen Z. (2021). Regulation and function of defense-related callose deposition in plants. IJMS 22:2393. doi: 10.3390/ijms22052393, PMID: 33673633 PMC7957820

[ref108] Wanjiru W. Zhensheng K. Buchenauer H. (2002). Importance of cell wall degrading enzymes produced by *Fusarium graminearum* during infection of wheat heads. Eur. J. Plant Pathol. 108, 803–810. doi: 10.1023/A:1020847216155

[ref109] Wei T. Tang Y. Jia P. Zeng Y. Wang B. Wu P.. (2021). A cotton lignin biosynthesis gene, *GhLAC4*, fine-tuned by ghr-miR397 modulates plant resistance against *Verticillium dahliae*. Front. Plant Sci. 12:3795. doi: 10.3389/fpls.2021.743795, PMID: 34868127 PMC8636836

[ref110] Wolf S. Mouille G. Pelloux J. (2009). Homogalacturonan methyl-esterification and plant development. Mol. Plant 2, 851–860. doi: 10.1093/mp/ssp066 19825662

[ref111] Wu D. Wang L. Zhang Y. Bai L. Yu F. (2021). Emerging roles of pathogen-secreted host mimics in plant disease development. Trends Parasitol. 37, 1082–1095. doi: 10.1016/j.pt.2021.09.007, PMID: 34627670

[ref112] Xiao S. Hu Q. Shen J. Liu S. Yang Z. Chen K.. (2021). *GhMYB4* downregulates lignin biosynthesis and enhances cotton resistance to *Verticillium dahliae*. Plant Cell Rep. 40, 735–751. doi: 10.1007/s00299-021-02672-x, PMID: 33638657

[ref113] Xie W. Ke Y. Cao J. Wang S. Yuan M. (2021). Knock out of transcription factor *WRKY53* thickens sclerenchyma cell walls, confers bacterial blight resistance. Plant Physiol. 187, 1746–1761. doi: 10.1093/plphys/kiab400, PMID: 34618083 PMC8566205

[ref114] Yang L. Ding W. Xu Y. Wu D. Li S. Chen J.. (2016). New insights into the antibacterial activity of hydroxycoumarins against *Ralstonia solanacearum*. Molecules 21:468. doi: 10.3390/molecules21040468, PMID: 27070570 PMC6273506

[ref115] Yang L. Wang Y. He X. Xiao Q. Han S. Jia Z.. (2021a). Discovery of a novel plant-derived agent against *Ralstonia solanacearum* by targeting the bacterial division protein FtsZ. Pestic. Biochem. Physiol. 177:104892. doi: 10.1016/j.pestbp.2021.104892, PMID: 34301354

[ref116] Yang L. Wei Z. Li S. Xiao R. Xu Q. Ran Y.. (2021b). Plant secondary metabolite, daphnetin reduces extracellular polysaccharides production and virulence factors of *Ralstonia solanacearum*. Pestic. Biochem. Physiol. 179:104948. doi: 10.1016/j.pestbp.2021.104948, PMID: 34802533

[ref117] Yang L. Wei Z. Valls M. Ding W. (2022). Metabolic profiling of resistant and susceptible tobaccos response incited by *Ralstonia pseudosolanacearum* causing bacterial wilt. Front. Plant Sci. 12:429. doi: 10.3389/fpls.2021.780429, PMID: 35069638 PMC8780990

[ref118] Yuan M. Jiang Z. Bi G. Nomura K. Liu M. Wang Y.. (2021). Pattern-recognition receptors are required for NLR-mediated plant immunity. Nature 592, 105–109. doi: 10.1038/s41586-021-03316-6, PMID: 33692546 PMC8016741

[ref119] Zeiss D. Mhlongo M. Tugizimana F. Steenkamp P. Dubery I. (2018). Comparative metabolic phenotyping of tomato (*Solanum lycopersicum*) for the identification of metabolic signatures in cultivars differing in resistance to *Ralstonia solanacearum*. IJMS 19:2558. doi: 10.3390/ijms19092558, PMID: 30158424 PMC6163672

[ref120] Zhang B. Gao Y. Zhang L. Zhou Y. (2021). The plant cell wall: biosynthesis, construction, and functions. J. Integr. Plant Biol. 63, 251–272. doi: 10.1111/jipb.13055, PMID: 33325153

[ref121] Zhang H. Hong Y. Huang L. Li D. Song F. (2016). *Arabidopsis AtERF014* acts as a dual regulator that differentially modulates immunity against *pseudomonas syringae* pv. Tomato and *Botrytis cinerea*. Sci. Rep. 6:251. doi: 10.1038/srep30251, PMID: 27445230 PMC4957219

[ref122] Zhao C. Wang H. Lu Y. Hu J. Qu L. Li Z.. (2019). Deep sequencing reveals early reprogramming of *Arabidopsis* root transcriptomes upon *Ralstonia solanacearum* infection. MPMI 32, 813–827. doi: 10.1094/mpmi-10-18-0268-r 31140930

[ref123] Zhao X. Mei W. Gong M. Zuo W. Bai H. Dai H. (2011). Antibacterial activity of the flavonoids from dalbergia odorifera on *Ralstonia solanacearum*. Molecules 16, 9775–9782. doi: 10.3390/molecules16129775, PMID: 22117168 PMC6264665

[ref124] Zhou X. Liao H. Chern M. Yin J. Chen Y. Wang J.. (2018). Loss of function of a rice TPR-domain RNA-binding protein confers broad-spectrum disease resistance. Proc. Natl. Acad. Sci. U. S. A. 115, 3174–3179. doi: 10.1073/pnas.1705927115, PMID: 29432165 PMC5866533

[ref125] Zuluaga A. P. Solé M. Lu H. Góngora-Castillo E. Vaillancourt B. Coll N.. (2015). Transcriptome responses to *Ralstonia solanacearum* infection in the roots of the wild potato *Solanum commersonii*. BMC Genomics 16:246. doi: 10.1186/s12864-015-1460-1, PMID: 25880642 PMC4391584

