# Supplementary material for: Induced defense strategies of plants against Ralstonia solanacearum
Source: Front Microbiol. 2023 Jan 26;14:1059799. doi: 10.3389/fmicb.2023.1059799 (PMC9910360; doi:10.3389/fmicb.2023.1059799)
Supplement: Supplementary file 1 [file Table_1.DOCX]

Table S1 Plant-*R. solanacearum* associated transcriptome data

| **Plant** | **Resistant** | **Susceptible** | | **Sample site** | **References** |
| --- | --- | --- | --- | --- | --- |
| Tobacco | 4411-3 | K326 | | Stem | (Pan et al., 2021) |
| Tobacco | Fandi3 | Yunyan87 | | Stem | (Li et al., 2021) |
| Tobacco | D101 | Changbohuang | | Roots | (Gao et al., 2019) |
| Tomatoes | Hawaii 7996 | West Virginia 700 | | Root | (French et al., 2018) |
| Tomato | Hawaii 7996 | Zhongnong No4 | | Root | (Wang et al., 2019) |
| Tomato | Hawaii 7996 | Zhongnong No4 | | Stem | (Wang et al., 2019) |
| Potato | F118 | F97 | | Root | (Zuluaga et al., 2015) |
| Potato | Helan 15 | | | Leaf | (Cao et al., 2020) |
| Ginger | mango ginger | ginger | | Leaf | (Snigdha and Prasath, 2021) |
| Peanut | J04 | J62 | | Root | (Chen et al., 2014) |
| Eggplant | E-31 | E-32 | | Stem | (Chen et al., 2018) |
| Arabidopsis | Col-0 and ABA receptor mutants (12458 and 112458) | | | Root | (Zhao et al., 2019) |
| Capsicum | KC350 | | Chilbok | Root | (HWANG et al., 2011) |

**REFERENCES**

| Cao, W., Gan, L., Shang, K., Wang, C., Song, Y., Liu, H., et al. (2020). Global transcriptome analyses reveal the molecular signatures in the early response of potato (*Solanum tuberosum* L.) to *Phytophthora* infestans, *Ralstonia solanacearum*, and Potato virus Y infection. *Planta* 252. doi: 10.1007/s00425-020-03471-6. |  |
| --- | --- |
| Chen, N., Yu, B., Dong, R., Lei, J., Chen, C., and Cao, B. (2018). RNA-Seq-derived identification of differential transcription in the eggplant *(Solanum melongena*) following inoculation with bacterial wilt. *Gene* 644, 137–147. doi: 10.1016/j.gene.2017.11.003. |  |
| Chen, Y., Ren, X., Zhou, X., Huang, L., Yan, L., Lei, Y., et al. (2014). Dynamics in the resistant and susceptible peanut (*Arachis hypogaea* L.) root transcriptome on infection with the *Ralstonia solanacearum*. *BMC Genomics* 15, 1078. doi: 10.1186/1471-2164-15-1078. |  |
| French, E., Kim, B.-S., Rivera-Zuluaga, K., and Iyer-Pascuzzi, A. S. (2018). Whole root transcriptomic analysis suggests a role for auxin pathways in resistance to *Ralstonia solanacearum* in Tomato. *MPMI* 31, 432–444. doi: 10.1094/mpmi-08-17-0209-r. |  |
| Gao, W., Chen, R., Pan, M., Tang, W., Lan, T., Huang, L., et al. (2019). Early transcriptional response of seedling roots to *Ralstonia solanacearum* in tobacco (*Nicotiana tabacum* L.). *Eur J Plant Pathol* 155, 527–536. doi: 10.1007/s10658-019-01788-x. |  |
| Hwang, J., Choi, Y., Kang, J., Kim, S., Cho, M., Mihalte, L., et al. (2011). Microarray analysis of the transcriptome for bacterial wilt resistance in pepper (*Capsicum annuum* L.). *Not Bot Hort Agrobot Cluj* 39, 49. doi: 10.15835/nbha3926820. |  |
| Li, Y., Wang, L., Sun, G., Li, X., Chen, Z., Feng, J., et al. (2021). Digital gene expression analysis of the response to *Ralstonia solanacearum* between resistant and susceptible tobacco varieties. *Sci Rep* 11. doi: 10.1038/s41598-021-82576-8. |  |
| Pan, X., Chen, J., Yang, A., Yuan, Q., Zhao, W., Xu, T., et al. (2021). Comparative transcriptome profiling reveals defense-related genes against *Ralstonia solanacearum* infection in tobacco. *Front. Plant Sci.* 12. doi: 10.3389/fpls.2021.767882. |  |
| Snigdha, M., and Prasath, D. (2021). Transcriptomic analysis to reveal the differentially expressed miRNA targets and their miRNAs in response to *Ralstonia solanacearum* in ginger species. *BMC Plant Biol* 21. doi: 10.1186/s12870-021-03108-0. |  |
| Wang, G., Kong, J., Cui, D., Zhao, H., Niu, Y., Xu, M., et al. (2019). Resistance against *Ralstonia solanacearum* in tomato depends on the methionine cycle and the γ‐aminobutyric acid metabolic pathway. *Plant J* 97, 1032–1047. doi: 10.1111/tpj.14175. |  |
| Zhao, C., Wang, H., Lu, Y., Hu, J., Qu, L., Li, Z., et al. (2019). Deep sequencing reveals early reprogramming of arabidopsis root transcriptomes upon *Ralstonia solanacearum* infection. *MPMI* 32, 813–827. doi: 10.1094/mpmi-10-18-0268-r. |  |
| Zuluaga, A. P., Solé, M., Lu, H., Góngora-Castillo, E., Vaillancourt, B., Coll, N., et al. (2015). Transcriptome responses to *Ralstonia solanacearum* infection in the roots of the wild potato *Solanum commersonii*. *BMC Genomics* 16. doi: 10.1186/s12864-015-1460-1. |  |
